# Risk factors for non-melanoma skin cancer development in renal transplant recipients: a 40-year retrospective study in Croatia

**DOI:** 10.3325/cmj.2022.63.148

**Published:** 2022-04

**Authors:** Nikolina Bašić-Jukić, Tajana Borlinić, Deša Tešanović, Ivica Mokos, Ivan Krešimir Lukić, Zrinka Bukvić Mokos

**Affiliations:** 1 Department of Nephrology, Arterial Hypertension, Dialysis and Transplantation, University Hospital Center Zagreb, University of Zagreb School of Medicine, Zagreb, Croatia; 2 Čakovec County Hospital, Čakovec, Croatia; 3 Department of Nursing, University North, Varaždin, Croatia; 4 Special Hospital Agram, Zagreb, Croatia; 5 Department of Urology, University Hospital Center Zagreb, Zagreb, Croatia; 6 Study of Nursing, Bjelovar University of Applied Sciences, Bjelovar, Croatia; 7 Department of Dermatology and Venereology, University of Zagreb School of Medicine, University Hospital Center Zagreb, Zagreb, Croatia

## Abstract

**Aim:**

To determine the prevalence of non-melanoma skin cancer (NMSC) and disease-specific risk factors in renal transplant recipients (RTRs).

**Methods:**

This retrospective cohort study enrolled 1232 RTRs (736 men) treated in University Hospital Center Zagreb over 40 years. The effect of sex, age at transplantation, geographic residence, dialysis vintage, and the type of immunosuppressive therapy on NMSC occurrence was investigated.

**Results:**

The prevalence of NMSC was 6.81%. Overall, 60.7% of patients developed basal cell carcinoma (BCC) and 30.9% of patients developed cutaneous squamous cell carcinoma (cSCC). Only 8.3% developed both tumors. The BCC:cSCC ratio was 1.76:1. The risk for NMSC was 50% higher in men. Patients older than 50 years at transplantation were at greater risk for NMSC development. Residence in an area with higher ultraviolet exposure and dialysis vintage before transplantation did not influence NMSC development. Cyclosporine and azathioprine treatment conferred a greater risk for NMSC than tacrolimus or mycophenolate mofetil treatment.

**Conclusion:**

RTRs are at high risk for NMSC development. Sex, age at transplantation, and type of immunosuppressive therapy play a role in tumor development.

Non-melanoma skin cancer (NMSC) is the most common human malignancy. It includes basal cell carcinoma (BCC) (75% of NMSC), squamous cell carcinoma (cSCC) (20% of NMSC), and several rare types of NMSC.

BCC is a slow-growing malignant epidermal tumor with low metastatic potential. Although epidemiological data are lacking due to the tumor multiplicity and underreporting in national cancer registers, over the last 15 years, increased BCC incidence has been observed (1,2). Risk factors for BCC development include genetic predisposition, Fitzpatrick skin type I and II, multiple sunburns in younger age, older age, male sex, immunosuppressive therapy, sun exposure, and exposure to different carcinogens (1-3). In organ-transplant recipients, due to the life-long need for immunosuppressive therapy, the risk of BCC is ten times higher than in persons who do not receive immunosuppressive therapy (3).

Cutaneous squamous cell carcinoma (cSCC) is also a malignant tumor of epidermal keratocytes, with local, regional, and distant metastatic potential. Risk factors are similar to those for BCC, with the addition of chronic wounds and burn scars. The most significant risk factor is long-term exposure to ultraviolet (UV) radiation (4). Regarding immunosuppressive therapy, cyclosporine-treated and azathioprine-treated patients have increased risk for cSCC development. The risk depends on age, duration and intensity of immunosuppression, and pre-existent skin damage (UV radiation) (5,6).

The population of renal transplant recipients (RTRs) is under constant risk for NMSC development and requires specific preventive measures. Both types of NMSC occur more frequently after kidney transplantation; the risk for cSCC development is 65-250 times greater than in non-transplant patients (5,6).

Data on NMSC prevalence and its specific risk factors in Croatian RTRs are lacking. To this end, we conducted an epidemiological study at the Croatian Referral Transplant Center to investigate whether the type of immunosuppression, older age at transplantation, and dialysis vintage were risk factors for NMSC development in our population. We wanted to identify the group of patients with end-stage renal disease who were under greater risk for NMSC development to closely monitor them after transplantation, to prevent cancer occurrence and treat NMSC in the early stage of development.

## PATIENTS AND METHODS

We conducted a retrospective study at the largest kidney transplantation center in Croatia, University Hospital Centre Zagreb. The study enrolled all the patients who underwent kidney transplantation due to end-stage renal disease (ESRD) since the beginning of transplantation in 1974 to 2014. Pediatric RTRs and patients who were transplanted in University Hospital Center Zagreb but were not monitored there afterward were not included.

From the hospital database, we extracted data on sex, current residence, dialysis vintage, age at transplantation, type of NMSC, and post-transplant immunosuppressive therapy protocol. Different immunosuppressive therapy protocols tailored for each patient were used, including calcineurin inhibitors (cyclosporine or tacrolimus), immunosuppressive drugs (azathioprine or mycophenolate mofetil), and corticosteroid therapy (prednisone). New immunosuppressants, mTOR inhibitors (sirolimus or everolimus), were also used, replacing cyclosporine or azathioprine, mainly due to malignant tumors acquired before or after transplantation.

Regarding current residence, the patients were divided into those living in coastal Croatia and those living in continental Croatia. The border between coastal and continental Croatia was set to the south border of the Karlovac County. In this way, we aimed to get an insight into NMSC incidence in the region with higher UV light exposure.

This research complied with the Declaration of Helsinki and was approved by the University Hospital Centre Zagreb Ethics Committee and the School of Medicine University of Zagreb Ethics Committee.

### Statistical analysis

Data were summarized as proportions, and medians (with interquartile range) or arithmetic means (with standard deviations). The groups were compared with the χ^2^ test or Wilcoxon's test. For differences in cancer development according to residence and immunosuppressive therapy regimen, we calculated the odds ratio (OR) with a 95% confidence interval (CI). The time to onset of cancer after transplantation was plotted by Kaplan-Meier curves, and the curves were compared with the log-rank test. The level of significance was set at *P* < 0.05. Data were analyzed with Microsoft Excel 2016 (Microsoft Corporation, Redmond, WA, USA) and R programming language (7).

## RESULTS

The study enrolled 1232 RTRs; 736 (59.74%) men. The overall NMSC incidence was 6.81% (n = 84), with 5.28% developing only one NMSC and the rest developing multiple NMSC. The occurrence ratio of BCC:cSCC was 1.76:1.

**Figure 1:**
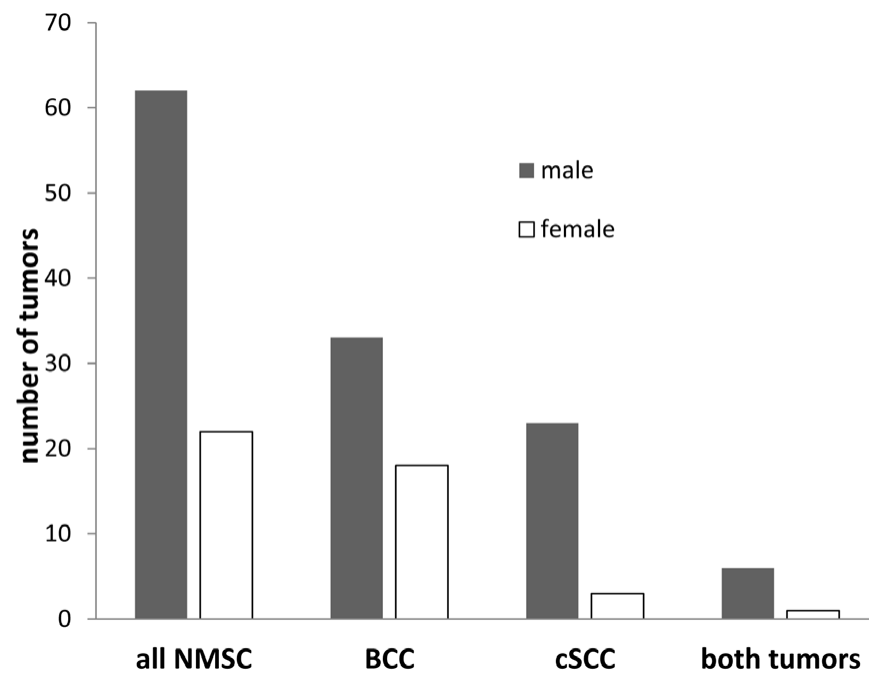
Non-melanoma skin cancer (NMSC), basal cell carcinoma (BCC) and cutaneous squamous cell carcinoma (cSCC), among male and female renal transplant recipients at Zagreb University Hospital Center from 1974 to 2014.

**Figure 2:**
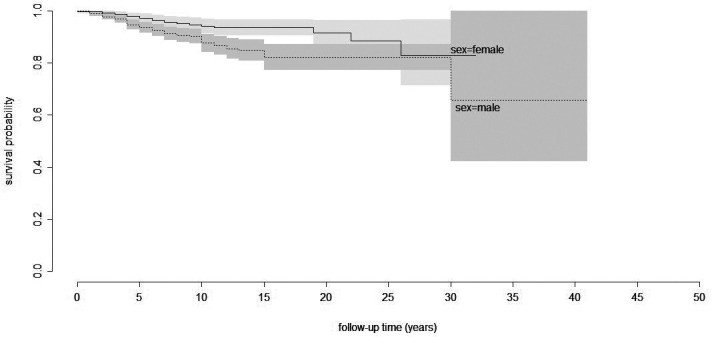
Time from transplantation to development of non-melanoma skin cancer in Croatian female and male renal transplant recipients. Shaded area: 95% confidence intervals. P (female vs male) = 0.002, χ^2^(log rank) = 9.5.

Among RTRS who developed NMSC, 73.8% were men with incidence ratio 2.6:1 (χ^2^, *P* = 0.007) (Figure 1). BCC was present in 33 men and 18 women, while cSCC was present in 23 men and 3 women. Men more frequently developed NMSC (χ^2^, *P* = 0.007). Seven patients (6 men and 1 woman) developed both tumors. Furthermore, the time from transplantation to the appearance of the first NMSC was significantly shorter in men (log rank test, *P* = 0.002) (Figure 2). RTRs who developed NMSC were significantly older than those who did not develop it (53.6 ± 10 years vs 46.0 ± 14.5 years; Wilcoxon test *P* ≤ 0.001). The median time from transplantation to the development of the first tumor was 5 (3-9.75) years.

Three hundred patients resided in the coastal part of Croatia, while the rest (n = 932) lived in the continental part. The two groups did not significantly differ in NMSC appearance (7.3% in continental and 5.3% in coastal Croatia; χ^2^ test, *P* = 0.241; OR 1.397, 95% CI 0.797-2.448).

**Figure 3:**
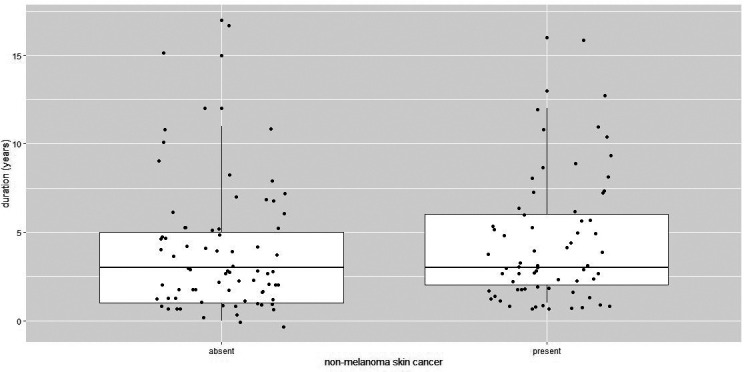
Dialysis vintage before transplantation in renal transplant recipients treated at the University Hospital Center Zagreb 1974-2014 who developed non-melanoma skin cancer (NMSC) after transplantation and patients who did not develop NMSC. Wilcoxon's test *P* = 0.272.

The median dialysis vintage in our RTRs was three years, and it did not affect the development of NMSC (Wilcoxon test, *P* = 0.272) (Figure 3).

**Figure 4:**
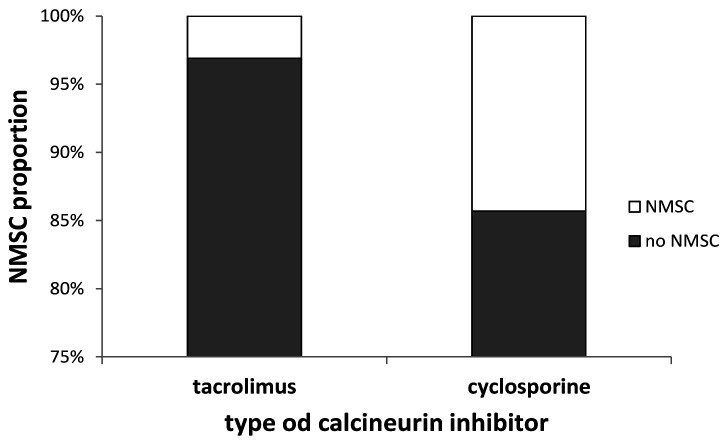
Proportion of Croatian renal transplant recipients treated at Zagreb University Hospital Center 1974-2014 who developed non-melanoma skin cancer (NMSC) while being immunosuppressed by cyclosporin as the main immunosuppressant compared with tacrolimus. χ^2^ test *P* < 0.001; odds ratio 0.19 (95% confidence interval 0.12-0.31).

**Figure 5:**
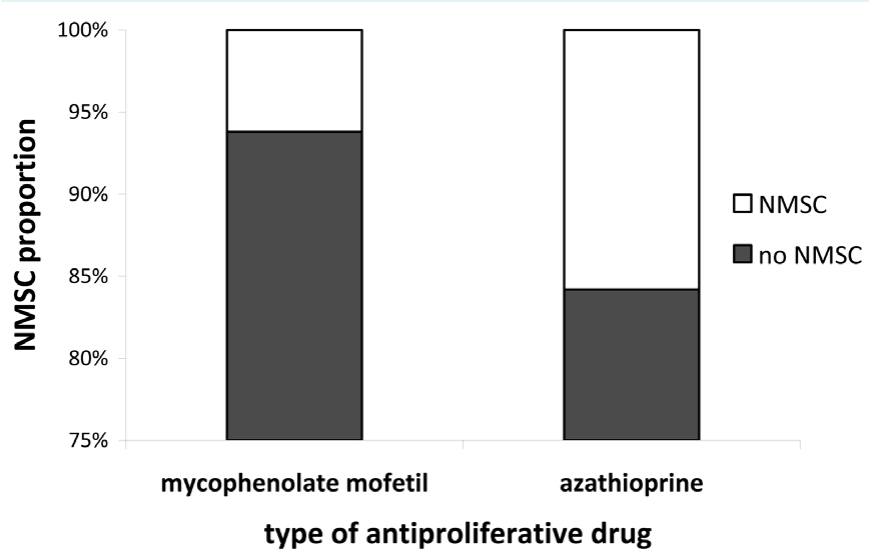
Proportion of Croatian renal transplant recipients treated at Zagreb University Hospital Center 1974-2014 who developed non-melanoma skin cancer (NMSC) while being immunosuppressed by mycophenolate mofetil compared with azathioprine. χ^2^ test *P* = 0.001; odds ratio 0.34 (95% confidence interval 0.18-0.62).

Significantly more patients treated with cyclosporine developed at least one NMSC compared with patients immunosuppressed by tacrolimus (14.3% vs 3.1%; χ^2^ test, *P* = <0.001). The relative risk for NMSC development while taking tacrolimus was OR 0.19 (95% CI 0.12-0.31) (Figure 4). Significantly fewer patients treated with mycophenolate mofetil (5.9%) developed NMSC compared with patients treated with azathioprine (15.8%; OR 0.34, 95% CI 0.18-0.62; χ^2^ test, *P* = 0.001) (Figure 5). Of patients who were receiving mTOR inhibitors, 4.2% developed NMSC.

## DISCUSSION

In this study, RTRs were at high risk for NMSC development. Sex, age at transplantation, and type of immunosuppressive therapy played a role in tumor development.

Relevant epidemiological data indicate that NMSC is the most common cancer in RTRs, which poses a substantial clinical and health care problem (5,6,8-10). Dermatological screening and identification of risk factors may help decrease the incidence rates of NMSC in RTRs (11).

A recent systematic review and meta-analysis showed the incidence of NMSC among RTR_S_ to be as high as 12.6%, with significant heterogeneity among countries (12). The incidence was highest in Australia (39.1%) and lowest in the Middle East (1.2%). The leading cause of these differences might be differences in the intensity of UV radiation; however, genetic factors and differences in sun-protection behaviors are potential risk factors too (12). The incidence of NMSC in our Center of 6.81% is comparable with that in European countries: 2.2% in Greece, 5.1% in Poland, and 5.1%, in the UK (8-10) but is significantly lower than in the Mediterranean part of Spain, Australia, and Mexico (25.3%, 28.1%, and 33.3%, respectively) (13,14).

In our study, the BCC:cSCC incidence ratio was 1.76:1. Although most clinical studies in RTRs pointed to a higher incidence of cSCC than of BCC (10,15-18), some studies showed a higher incidence of BCC (5,13,19). The incidence of cSCC was higher in studies from Mexico, Brazil, Australia, and some European countries (6,8,10,14,16-18), while BCC was more frequent in the Mediterranean part of Europe (5,9,13,19). Higher BCC prevalence in our RTRs might also be explained by a high-level screening of patients before and after transplantation in our Center, whereby precancerous lesions (cSCC precursors) are diagnosed and treated early.

Some researchers indicated male sex as a risk factor for the development of NMSC in RTRs (16,20). We confirmed these findings, showing that male RTRs developed NMSC almost three times more frequently than female RTRs. Furthermore, the time from kidney transplantation to the appearance of the first tumor was much shorter in men. Higher tumor incidence and shorter time to tumor development in men could be explained by men less frequently using sun protection (21-23). Therefore, further efforts should be made in education, especially among RTRs, about the need and benefits of using sun-protection to prevent NMSC.

We noticed that the RTRs who developed NMSC were on average eight years older at transplantation than those who did not develop NMSC, which indicates that older age at transplantation is one of the risk factors for the development of NMSC. Our results agree with previous research suggesting that older age at transplantation (>50 years old) was a risk factor for NMSC development (8,10,13).

The time from transplantation to the first tumor occurrence of 5 years in our study is consistent with previous results (9,14,16). However, most recent studies indicate that this period was shortening and was as short as 3.75 years (13,24). The cause for earlier tumor development after transplantation might be the aging population in general and the increased number of patients who had their kidney transplantation after the age of 50 years, especially among Eurotransplant members. Eurotransplant matches organ donors and ESRD patients, thus enabling transplantation in more patients.

Sun exposure, leisure or professional, is one of the major factors for NMSC development, not only in the general population but also in organ transplant recipients (13,14). The coastal part of Croatia annually receives longer and more intensive sun exposure than the continental part (4681-5760 MJ/m^2^ and 3161-4680 MJ/m^2^, respectively) (25). However, we found no significant difference in NMSC occurrence between the RTR population of coastal and that of continental Croatia. This result might be attributed to the frequent migration of the Croatian population during their life span from the coastal to continental regions and increased professional sun exposure in continental Croatia.

Although some studies found that longer dialysis vintage before kidney transplantation negatively affected clinical outcomes, and that preemptive kidney transplantation should be beneficial (26), others found no effect of dialysis vintage on NMSC development (13). In our research, dialysis vintage did not affect NMSC development. The median of dialysis vintage of three years before the kidney transplant was the same among the patients with NMSC and those without NMSC, and it is similar to previous research (13,16). Nevertheless, since Croatia became a member of Eurotransplant in 2010, waiting lists for kidney transplants significantly shortened; some patients had their kidney transplantation even without pre-treatment by dialysis (preemptive). Future research is expected to evaluate whether shorter dialysis vintage will influence NMSC development.

In our study, cyclosporine-treated patients had an 80% higher risk of NMSC development compared with tacrolimus-treated patients, whereas patients treated with azathioprine had a 60% higher risk of NMSC development compared with mycophenolate mofetil-treated ones. Cyclosporine inhibits UVB-induced DNA damage repair, thus facilitating skin carcinogenesis (27). Therefore, a significantly higher risk of NMSC development in cyclosporine-treated patients might be due to the long-term cyclosporine therapy, which in some patients lasted over 30 years.

In this regard, our results agree with previous studies. Krásová et al (28) found a significantly increased risk of NMSC development in patients treated with cyclosporine compared with tacrolimus, mycophenolate mofetil, steroids, and their combinations. Cyclosporine substitution by mTOR inhibitor decreased the incidence of NMSC, while even only adding sirolimus to cyclosporine therapy slowed down tumor growth and progression (29,30).

As for azathioprine, Perret et al (31) observed that azathioprine-treated patients showed increased UVA sensitivity. In addition, azathioprine-treated patients had a two times higher risk of NMSC development (32). Hofbauer et al (33) showed twice lower minimal erythema dose (MED) for UVA rays during azathioprine therapy than during mycophenolate mofetil (an antiproliferative drug) therapy, which then enhanced UV-induced carcinogenesis in these patients.

Recent studies indicate that mTOR inhibitors, especially sirolimus, inhibit tumor development in multiple ways that are not fully understood. It seems that they reduce skin tumor progression and UV ray-induced mutations (30). In our Center, tacrolimus has been the first-choice calcineurin inhibitor since 2009. Our patients were switched to mTOR inhibitor therapy, mainly due to malignant tumors acquired before or after transplantation. From these patients, 4.2% developed NMSC, indicating that immunosuppression by mTOR inhibitors did not protect them from NMSC development. Considering the small number of patients who received mTOR inhibitors (n = 144) and different combinations of immunosuppressants, it was not possible to evaluate the significance of NMSC incidence in patients receiving this therapy. Patients have to be followed up further to adequately conclude on the relation between mTOR inhibitors and NMSC development. A recent meta-analysis in non-renal transplant recipients indicates that mTOR inhibitors may prevent second NMSC development but do not influence the development of the first skin cancer (34).

Our study included the largest cohort of RTRs in Croatia. It is the first research focusing on risk factors for NMSC development in RTRs in Croatia. However, it suffers from several limitations. First, this is a single-center study, and the results may not be representative of other patients in Croatia. Nevertheless, University Hospital Center Zagreb is the largest national kidney transplant center with a transplantation history of more than 40 years, treating a vast majority of Croatian patients. Since there is no national registry of RTRs in Croatia, our data are valuable for predicting NMSC development in our population. Second, all patients were not regularly dermatologically assessed, despite the recommendations from nephrologists. Finally, patients treated with cyclosporine had significantly longer exposure to immunosuppression, which may be a source of bias.

In conclusion, our results showed that posttransplant immunosuppression with tacrolimus and mycophenolate mofetil carried a lesser risk for NMSC development than immunosuppression with cyclosporine and azathioprine. Therefore, we suggest that tacrolimus and mycophenolate, together with mTOR inhibitors, should be the treatment of choice in RTRs. Male patients with end-stage renal disease and patients older at transplantation were at higher risk for NMSC development after transplantation. Therefore, they should be more carefully monitored after transplantation.

Since the number of patients transplanted after 50 years of age is rising, and the time to first tumor development is getting shorter, we suggest that all patients with end-stage renal disease should be referred to dermatological assessment before transplantation and regularly monitored after transplantation in order to prevent NMSC or treat it as early as possible.
